# Transition to middle school: Self-concept changes

**DOI:** 10.1371/journal.pone.0212640

**Published:** 2019-02-20

**Authors:** Wanesa Onetti, José Carlos Fernández-García, Alfonso Castillo-Rodríguez

**Affiliations:** 1 UNIR, International University of La Rioja, Faculty of Education, Logroño, Spain; 2 Department of the Languages, Arts and Sport Didactic, Universidad de Malaga, Andalucía-Tech, IBIMA, Malaga, Spain; 3 Department of Physical Education and Sports, University of Granada, Granada, Spain; Universidad Nacional de Educacion a Distancia (UNED), SPAIN

## Abstract

Self-concept influences identity and the way that people behave, and it fluctuates over time. The main purpose of this study was to analyze fluctuations in the dimensions of self-concept as a function of gender, educational level, grade, age, physical activity, and weight. In total, 712 Spanish adolescents who were in the 5^th^ and 8^th^ grades (354 boys and 358 girls) and 10 to 14 years old (*M* = 11.9; *SD* = 1.3) participated in this study. The Self-Concept Questionnaire, Form 5 was used to analyze several dimensions of self-concept (academic, social, emotional, family, and physical), using the average scores in each dimension. The data showed strong differences in the dimensions of self-concept during the school transition. Middle-school students, compared to elementary-school students, showed significantly lower levels in almost all dimensions (academic, social, family, and physical). Furthermore, student age was a negative predictor of the social and academic dimensions, explaining 33% and 37% of the variance, respectively. Educational level and grade were smaller factors influencing the academic dimension (explaining 29% and 25% of the variance, respectively). The main findings revealed that the school transition and, specifically, increased age were associated with a lower self-concept. These results help us understand the need to strengthen psychological and educational self-concept at school.

## Introduction

Self-concept is a psychosocial variable that evaluates the perception an individual has of herself [1], and it has been established as one of the most important constructs in the social sciences and as fundamental to psychological wellbeing [2]. Children’s mental and cognitive health is associated directly and positively with an active physical lifestyle [3]. Specially, self-concept and self-sufficiency are positively associated with physical condition [4, 5, 6]. In general, a better physical condition predicts a better self-concept [3, 7, 8].

### Self-concept implications

An adequate self-esteem facilitates engagement in school activities, social skills, leadership, motivation and goal attainment. Consequently, it is propitious to a positive self-concept. Personality disorders have a detrimental effect on self-concept. In addition, children are at risk for other kinds of physical and psychosocial problems [9], in both the short and long term [10, 11], due to the physical inactivity associated with a sedentary lifestyle, which is so common in developed societies [10].

In studies carried out on groups of adolescents, it has been verified that physical activity has a positive influence on the development of the physical dimension of self-concept [9], and other studies done by Alfermann and Stoll [12], Fox [13] and Velez, Golem, and Arent [6] showed that physical activity in this population is beneficial for the physical, physiological, and most notably, the psychological aspects of people.

### Fluctuation in self-concept

With regard to childhood, it is known that self-concept plays a large role in the construction of personal identity. Recently, Saad, Damien, Benet-Martínez, Moons, and Robins [14] stated that self-concept has various dimensions that explain the different aspects of a person, e.g., his appearance or physical image, his physical capacities, his different psychological characteristics, his social relationships, etc. As noted by models presented by Bronfenbrenner [15] and Cunha and Heckman [16], parenting behavior and home, child care, and neighborhood characteristics are likely to influence young children’s academic, social, and emotional dimensions of self-concept [17]. Overall, Pesu, Viljaranta, and Aunola [18] have confirmed that, during first grade, it is teachers’ rather than parents’ beliefs that play a role in children’s self-concept of ability. As such, they recommended the importance of supporting children’s developing self-concept as well as teaching new academic skills.

A child, from age seven, begins to worry about his body image and his psychosocial behavior and may develop disturbing attitudes about eating [19, 20]. In this respect, girls have a more positive perception of themselves during childhood, although, after the age of 12, their self-confidence declines [21]. This decline seems to be due to several factors [22]. First, there are findings that boys often receive preferential treatment from their teachers during school hours [21]. Second, according to Ahern [23], globally, girls are at higher risk than boys and men for many psychological disorders, particularly depression and anxiety. This gender difference in depression may be associated with a difference in coping styles as, generally speaking, girl adolescents are more likely to ruminate in response to stress, and ruminating has been linked to depression [24], which may explain the decline in their self-image [25]. In other words, it appears that there exist differences in self-image in relation to sex [25] and age [21].

### Middle school transition

The middle school (MS) transition causes relevant changes in adolescents [26] during their passage through different grades [27], e.g., psychological and emotional alterations [28, 29, 30]. Lerner, Bowers, Geldhof, Gestsdóttir, and DeSouza [31] confirmed that this school transition produces important negative changes, such as stress, social problems (friends and / or family), and physical changes, among others, that affect adolescents’ development of personality. In addition, Molloy, Ram, and Gest [32] found a decrease in academic and social self-concept during this transition. The hypothesis that we propose, for these reasons, is that self-concept decreases as students increase in age following the school transition, which is in contrast with the last year of elementary school (ES).

The development of self-concept is a key factor that corresponds with the willingness to achieve goals and overall well-being during the MS transition [2]. The aim of the school transition is reaching significant social and personal development in adulthood, which, previously, is necessary to perform the skills that facilitate and benefit this transition [33].

Therefore, education is important for the acceptance of oneself as a preventive tactic to achieve psychological adaptation in adolescence [34]. These authors have related positive self-perception during adolescence to different indicators of adaptation, supporting the idea that high self-concept is associated with better psychological adjustment and personal competence and fewer behavioral problems.

### Current study

These considerations prompted us to examine changes in the dimensions of self-concept among predominantly ES and MS public school students, aged 10 to 14 years, in fifth to eighth grade, comprising early adolescence (categorized by Sawyer et al. [35]). We refer to this sample of students according to their socioeconomic characteristics, being that the schools were chosen at random among different areas of the city in which the socioeconomic level was average. Therefore, for descriptive clarity, we refer to the sample as being composed of predominantly average students. That said, the first objective of this study was to analyze self-concept dimensions in Spanish students according to their sex, education level, grade, age, physical activity, and weight. The second objective was to assess the relationship between dimensions of self-concept and the physical and social-demographic characteristics of the students.

## Materials and methods

### Participants and research design

To recruit a sufficient number of participants, 10 different schools in a Spanish city were examined for this study. The selection of schools was made at random. Those selected were located in the downtown area in a middle class neighborhood. The following inclusion criteria were used to recruit students: Students must have been between 10 and 14 years old; they must have been enrolled in the 5^th^ through 8^th^ grade; they should have been able to read and answer the test questions. Furthermore, incomplete tests resulted in exclusion. Initially, a total of 800 students participated. However, 88 questionnaires were incomplete, with at least two questions unanswered.

Ultimately, 712 students from the south of Spain participated in this study; specifically, 354 boys and 358 girls. Their ages ranged from 10 to 14 years. Physical characteristics such as weight, height, age, and body mass index (BMI) can be found in Table 1. Four separate grades were analyzed: the fifth and sixth grades of ES and seventh and eighth grades of MS.

**Table 1:** T-test for physical characteristics according to the sex.

		Boys (*N* = 354)	Girls (*N* = 358)	*p*
Age	(years)	11.93 ± 1.41	11.76 ± 1.22	0.501
Weight	(kg)	47.74 ± 9.94	43.82 ± 10.3	0.050
Height	(m)	1.56 ± 0.15	1.52 ± 0.11	0.136
BMI	(kg·m^-2^)	19.33 ± 3.13	18.80 ± 2.79	0.223

BMI: Body Mass Index.

### Measures

#### Physical and socio-demographic characteristics

Participants completed an ad hoc questionnaire, with questions pertaining to age, sex, and socio-demographic characteristics such as grade, education level, physical activity and participation in sports. An electronic scale, SECA (Hamburg, Germany), was used to measure body weight to the nearest 100 g, and height was measured to the nearest 0.1 cm with a SECA electronic stadiometer (SECA Ltd, Medical Scales and Measurement Systems, Birmingham, United Kingdom). Using these measurements, we calculated overweight status via BMI according to Cole, Bellizzi, Flegal, and Dietz’s [36] classification system (e.g. 10-year-old boys weighing 18.6 kg·m^-2^ or more and 10-year-old girls weighing 19.1 kg·m^-2^ or more were considered overweight).

#### Self-concept dimensions

Self-Concept Questionnaire, Form 5 validated for Spanish sample [37](level of reliability = 0.85) is based on the theoretical model created by Shavelson, Hubner, & Stanton [38] and consists of 5 subscales, each one is measured by 6 items: academic/occupational (items 1, 6, 11, 16, 21, and 26), social (items 2, 7, 12, 17, 22, and 27), emotional (items 3, 8, 13, 18, 23, and 28), family (items 4, 9, 14, 19, 24, and 29), and physical (items 5, 10, 15, 20, 25, and 30). In this way, using a Likert-type scale from 1 to 99, one single instrument measured the self-concept dimensions [37]. In this study, the reliability level was of mean, α = 0.83, where the minimum value was in emotional dimension with α = 0.70.

### Procedure

A cross-sectional quantitative methodology was used to administer the Self-Concept Questionnaire, Form 5, along with socio-demographic and other questions, which were posed to identify the following for each participant: level of physical activity performed outside of school hours, age, sex, height, weight, and BMI. These data were collected at the 10 schools for 2 months.

Parents and guardians were briefed on the objective of the study and signed a voluntary informed consent form allowing their children to participate. This study was in compliance with the guidelines found in the Helsinki Declaration (2013), which establishes ethical principles for investigations using humans. The Ethics Committee from the University of Granada in Spain approved the implementation of this study (number 471/CEIH/2018). Additionally, during and after the entire research process, we proceeded under the provisions of Spanish Organic Law 15/1999 of December 13^th^ for the protection of personal data. All the participants were treated according to the ethical indications of respect, confidentiality and anonymity in the treatment of their data.

### Statistical analyses

Microsoft Excel 2010 (Microsoft Corp, Redmond, Washington, USA) and SPSS for Windows (SPSS Inc., Chicago), version 21.0 were used. First, Kolmogorov-Smirnov analyses were performed in order to test the dependent variables for normality. The results showed that these variables adhered to a normal distribution. Second, to perform descriptive analyses of the dependent variables, comparative analyses (T-test and ANOVA test) were conducted using the independent variables of sex, age, physical activity, and educational and overweight levels [36], and a correlational analysis (Pearson test) was conducted. Third, a linear regression analysis (stepwise method) was performed modeling the relationship between physical characteristics and the dimensions of self-concept. Standardized units equivalent to 0.20 (that is, a fraction of the standard deviation between participants at the beginning of this research) were selected as the smallest significant change [39]. The size of the effect (*η*^*2*^) was used to quantify the size of the difference that was found between both groups. According to this, we could say that this is a true measure of the significance for such a difference [40]. The threshold values for the Cohen effect sizes detected in a t-test (*d*) are 0.20 for small effects, 0.50 for moderate effects, and 0.80 for large effects. For ANOVA tests, those values (*η*^*2*^) were 0.10 for small effects, 0.25 for moderate effects, and 0.40 for large effects. For multiple regression analysis, those values (ƒ^2^) were 0.02 for small effects, 0.15 for moderate effects, and 0.35 for large effects. The level of significance was *p* < 0.05.

## Results

### Comparison of the self-concept dimensions among physical characteristics

The data revealed few sex differences in the self-concept dimensions. Girls exhibited significantly higher values for the emotional dimension (*M* = 2.55, *SD* = 2.14 versus *M* = 3.53, *SD* = 2.24; *t*(701) = -2.329; *p* = 0.010; *d* = 0.22 [medium]) than did boys. Next, differences in the self-concept dimensions according to overweight status, as determined by BMI, were analyzed. All of the dimensions were similar across the overweight and normal weight adolescents. Normal-weight adolescents had higher scores for all dimensions than overweight adolescents, but these differences were small (*p* > 0.05). Table 2 shows the differences in the self-concept dimensions depending on age. Academic, emotional and family dimensions were significantly different according to age. Specifically, the 14-year-old participants had lower scores for the academic and family dimensions (*p* < 0.001; *η*^*2*^ = 0.42 and 0.23 [medium and small, respectively]), and the 10-year-old participants had lower scores for the emotional dimension (*p* = 0.050; *η*^*2*^ = 0.14 [small]).

**Table 2:** ANOVA test for self-concept dimensions according to the age.

	10-year-olds (*N* = 137)	11-year-olds (*N* = 155)	12-year-olds (*N* = 146)	13-year-olds (*N* = 140)	14-year-olds (*N* = 134)	*F*_*(1*,*711)*_	*p*	*η*^*2*^
Academic	8.89 ± 1.09^**13,14**^	8.98 ± 0.98^**13,14**^	8.15 ± 1.72^**14**^	7.08 ± 2.06^**10,11,14**^	4.86 ± 3.12^**10,11,12,13**^	21.325	0.000	0.42
Social	9.01 ± 0.96	8.53 ± 1.76	8.72 ± 1.19	8.28 ± 1.66	7.49 ± 2.60	2.121	0.061	0.14
Emotional	2.24 ± 2.00^**13**^	2.69 ± 2.27	3.58 ± 2.06	5.99 ± 2.18^**10**^	2.85 ± 2.67	2.271	0.050	0.14
Family	9.00 ± 1.29	9.54 ± 0.54^**14**^	9.39 ± 0.79^**14**^	8.76 ± 1.62	7.76 ± 2.62^**11,12**^	6.352	0.000	0.23
Physical	8.35 ± 1.12	8.28 ± 1.94	7.49 ± 1.40	6.89 ± 2.56	7.23 ± 3.10	2.021	0.082	0.13

Bonferroni post hoc (*P* < 0.05); Exponent number indicates the difference among to ages. *η*^*2*^: Effect size.

### Comparison of the self-concept dimensions during the school transition

**Fig 1:**
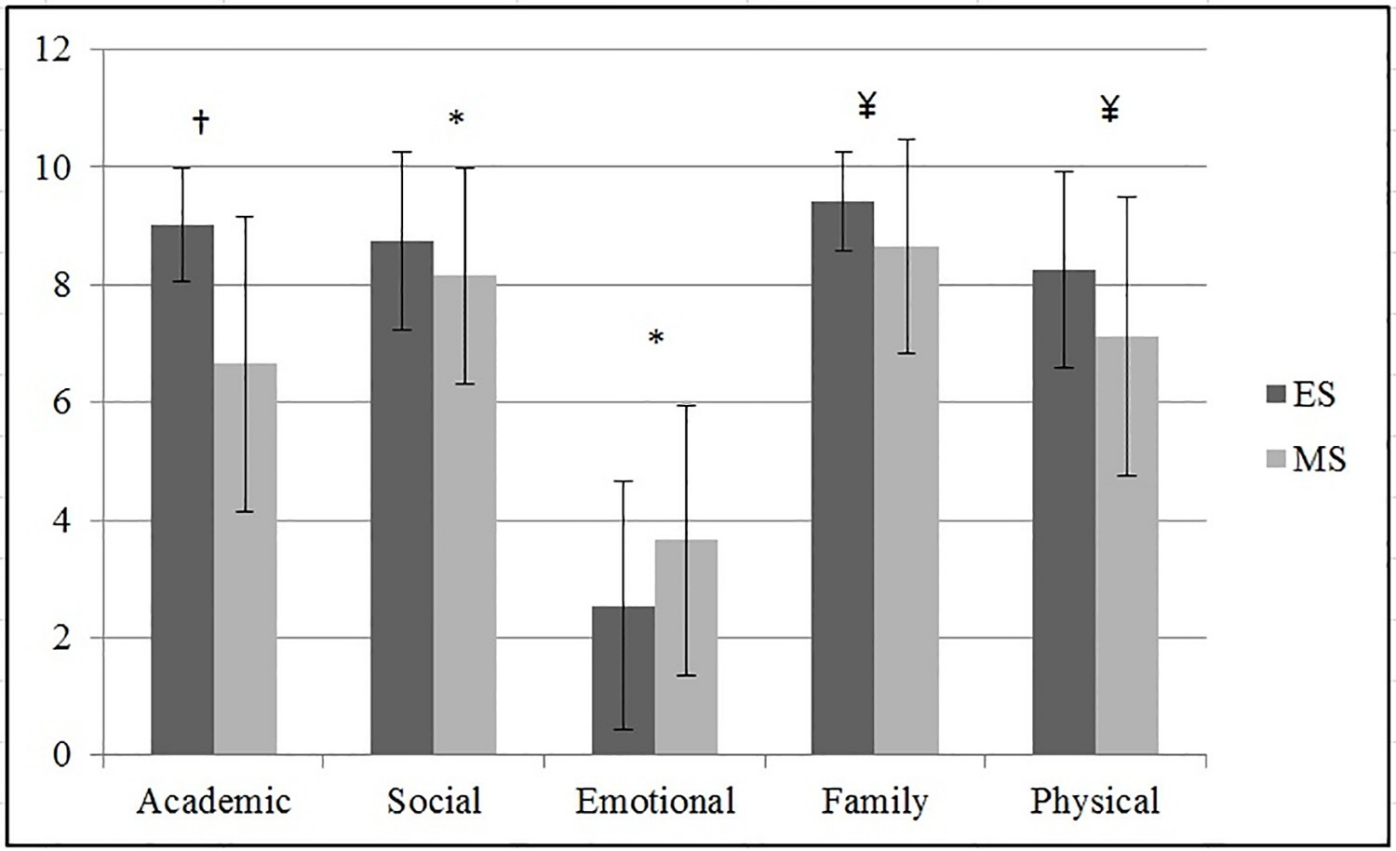
Differences of self-concept dimensions according to the educational level. ES: Elementary-School; MS: Middle-School; † *p* < 0.001, ¥ *p* < 0.01; * *p* < 0.05.

**Fig 2:**
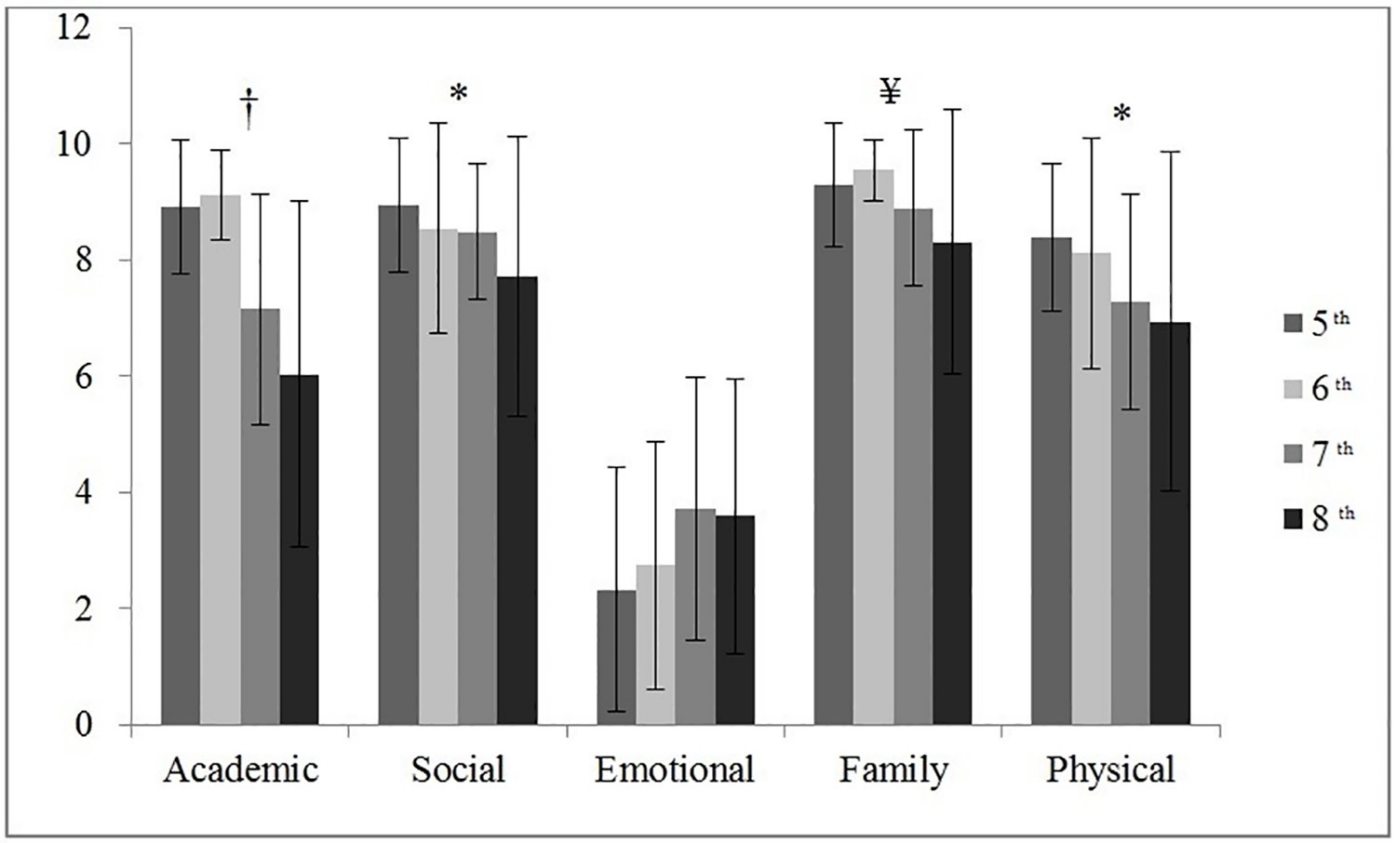
ANOVA test for self-concept dimensions according to the grades. † Differences 7^th^ and 8^th^ grades with 5^th^ and 6^th^ (*p* < 0.001); * Differences 8^th^ with 5^th^ grades (*p* < 0.05) ¥ Differences 8^th^ with 6^th^ grades (*p* < 0.05).

Figs 1 and 2 show the differences in self-concept based on educational level (ES or MS) and grade (5^th^ to 8^th^), respectively. ES students had higher values for the academic, social, family and physical self-concept dimensions (*p* < 0.05; *d* = 0.54, 0.18, 0.27, and 0.27 [large, small, and medium, respectively]) than MS students, who had a higher emotional self-concept (*M* = 3.66, *SD* = 2.29 versus *M* = 2.54 *SD* = 2.11; *p* < 0.05; *d* = 0.25 [medium]). On the other hand, students in the last two years of ES had a higher academic self-concept than students in the first two years of MS (*p* < 0.01; *η*^*2*^ = 0.33 [large]). Similarly, fifth grade students had a higher social and physical self-concept than eighth grade students (*p* < 0.05; *η*^*2*^ = 0.06 and 0.08 [small]). Lastly, sixth grade students had a significantly higher family self-concept than seventh and eighth grade students (*p* < 0.05; *η*^*2*^ = 0.10 [small]).

### Relationship between psychological and physical characteristics

Pearson correlation coefficients between the self-concept dimensions and the physical characteristics of the participants demonstrated varying degrees of inverse relationships: first, between the academic dimension and height and weight (*r* = -0.30 and -0.29; *p* < 0.01, respectively), second, between the academic dimension and age and educational level (*r* = -0.50 and -0.56; *p* < 0.01, respectively), and, third, between the physical self-concept dimension and age and educational level (*r* = ranging from -0.20 to -0.27; *p* < 0.05).

### Determinants of change in self-concept during school transition

Lastly, a multiple linear regression analysis (stepwise) was performed. According to this, the social and academic dimensions were significantly predicted by age (*R*^*2*^ = 0.33; *SEE* = 1.73; *p* < 0.01 and *R*^*2*^ = 0.37; *SEE* = 1.75; *p* < 0.01; respectively). The emotional dimension was predicted by the variables age, educational level, weight, and BMI (*R*^*2*^ = 0.26; *SEE* = 1.98; *p* = 0.01; *f*^*2*^ = 0.30 [large]). The physical dimension was predicted by the variables BMI, sex, and grade (*R*^*2*^ = 0.14; *SEE* = 1.78; *p* = 0.02; *f*^*2*^ = 0.25 [large]).

## Discussion

The primary purpose of this study was to analyze the self-concept dimensions of ES and MS students according to their sex, age, grade, educational level, physical activity and overweight status. The secondary purpose was to explore the relationships between different self-concept dimensions and the socio-demographic and physical characteristics of the students. No differences were found between the sexes in relation to the self-concept dimensions, except for the emotional dimension. This absence of differences was congruent with results obtained by Amezcua-Membrilla and Pichardo-Martínez [22], Coelho, Marchante, and Jimerson [41], and Guillén and Ramírez [42]. The exception, the higher scores on the emotional dimension found among girls, corresponds with other studies [43, 44, 45]. These data assessed the school transition using the entire sample, without separating the participants into groups based on sex.

### What factors were associated with the self-concept dimensions?

There were large age differences between some self-concept dimensions, with lower self-concept levels among MS students than among ES students. From the age of 7, children have a high interest in their body image, although they still have no critical sense of their body, which occurs during puberty when adolescents are not always happy with their body [46], mainly due to social pressure towards a canon of stipulated beauty [47]. Therefore, it is important to consider how the self-concept dimensions change in children and adolescents [48]. Specifically, age was inversely related to the social dimension of self-concept. These results do not correspond with those of other studies, such as those by Guillén and Ramírez [42], who demonstrated higher levels of self-concept among older students (within ES]. This difference might be attributed to the exact ages of the participants, given that, in the named studies, the children were younger, and there was only one educational level (i.e. ES]. In the present study, significant differences were found in all of the self-concept dimensions between the ES and MS students (Fig 1).

Self-concept is a protective factor against psychological problems [49, 50]. An adequate self-concept for mental development is essential; therefore, it benefits a better school transition for adolescents [2]. In this transition, adolescents undergo important academic, physical, social and family changes [28, 29, 30], as demonstrated in this study. Our data suggest that, during the school transition, academic self-concept decreases due to an increase in academic demand [51], although it is also possibly due to changes in the relationship between teachers and students [52]. In addition, a reduction in physical self-concept was found as the adolescents aged [53] and experienced morphological changes associated with puberty. In the present study, adolescents experienced a decrease in social self-concept during the school transition. A combination of pubertal hormonal changes and multifaceted social stressors may cause early adolescents to be increasingly susceptible to wide mood swings, emotional instability and reduced impulse control [54]. Family support, in this sense, is especially important as a main agent of socialization [55]. The family self-concept is the best predictor of adolescent adaptation to his new more independent life, is also affected by this transition [56]. Therefore, psychologists and teachers must respond to this problem. When adolescents progress through the school transition period with guidance, strategies and proposals, they can modify their self-concept in order to achieve academic, social, family and physical well-being.

On the other hand, differences in the self-concept dimensions were not found according to overweight status and physical activity levels. Various studies claim that physical condition and body composition are related to cognitive functioning [57, 58, 59, 60] and that being overweight is related to a lower self-perception of physical ability and body image, which consequently affect psychological well-being and emotional state [9]. The present study found limited variance that could be attributed to the independent variable of overweight status, although, when BMI was added to the predictors of sex and grade, an inverse relationship between physical self-concept and these variables was found, explaining 14% of the variance. Crocker et al. [4] also analyzed the relationship of self-concept with body composition and physical condition. They studied Canadian adolescent girls, and found that BMI was inversely related to overall self-concept and to the physical dimension of self-concept. Although, for decades, physical activity has been purported to have positive effects on psychosocial well-being [61], our students did not differ depending on their physical activity because the students spent fewer hours practicing sports. This fact could be taken into account in order to examine the number of hours that students practice physical activity each week, being that a small number of hours may not have a positive effect on the psychological responses evaluated.

This study owns some shining points or highlights. First, this study assessed the change of the self-concept dimensions in Spanish students across the school transition of elementary to middle school and with other factors as age, sex, weight, height, physical activity, and grades. There are no other studies that have analyzed all the factors that have been included; therefore, it is presented as a multifaceted study. Second, some studies have evaluated the impact of age on self-concept, but none of them have contributed the percentage of the variance that predicts the fluctuation of self-concept through this factor. Third, there is a controversy among authors about the existence of differences in self-concept based on sex. Pursuant to the data of our study, girls have greater emotional self-concept than boys, but in general, there is a similarity of self-concept among boys and girls (incorporating age as a covariate in the statistical treatment). Finally, this study clarifies the linear evolution of self-concept dimensions according to academic courses from 5th grade to 8th grade.

### Limitations and future directions

Although the results of this study are of special interest from the point of view of pedagogical orientation and for psychologists working in schools because they could verify the effect of this transition, there are also some limitations. The classrooms selected for this study resulted in a sufficiently large sample to examine self-concept during the last two years of ES and the two years of MS. However, it would have been better to take into account the three years before the transition and the three years after in order to better establish changes in self-concept. It could be that with the next stage transition, adolescents experience new changes, mainly due to social and emotional processes. In general, to think that age can be associated with a decline in self-concept during a particular moment in life is very interesting. It would be valuable to undertake a longitudinal intervention study to assess or clarify the effect of a psycho-educational program on students. Future research projects in this topic could conduct a longitudinal study of the evolution of student and provide more information about the variance of self-concept and above all, the transition from the ES to the MS.

## Conclusions and practical implications

The main finding of this study reveals that self-concept decreases during the transition from ES to MS, and these differences increase as students move on to higher grade levels. Furthermore, it is inversely related to the age of the student, explaining 33% and 37% of the variance in social and academic self-concept, respectively. These findings suggest that the transition from ES to MS could be targeted to promote joint intervention programs among students so that these dimensions of self-concept do not decrease during this transition. Additionally, in order for all adolescents to gain access to organized activities at the school, these activities could be provided within the last two years of ES and the first two years of MS as both prevention and positive youth development programs.

## Supporting information

S1 File Minimal data set_PLosOne.sav. (SAV)
